# V-CHIMERA: An Immune-Inspired Verified Framework for Organizational Cyber Crisis Response Under Misinformation

**DOI:** 10.3390/biomimetics11050324

**Published:** 2026-05-06

**Authors:** Fahad Alghamdi, Saad Alqithami

**Affiliations:** Computer Science Department, Al-Baha University, Albaha 65779, Saudi Arabia; fghamdi@bu.edu.sa

**Keywords:** cyber incident response, crisis communication, misinformation, social cybersecurity, biomimetics, artificial immune systems, danger theory, multi-agent systems, runtime enforcement, socio-technical risk

## Abstract

In organizational cyber crises, incident response and official communication form coupled control loops, yet they are usually engineered separately. We present V-CHIMERA (Verified Coupled Human–Information–Machine Incident Response Architecture), a framework for organizational cyber crisis response under misinformation that jointly models cyber state, belief dynamics, trust, and communication governance. The framework combines three elements: an explicit cyber–social coupling architecture, a runtime protocol shield for communication safety, and immune-gated coupling (IGC) that uses danger signaling, tolerance thresholds, and immune memory to regulate when social feedback should affect operational response and how strongly counter-messaging should be targeted. Across three representative scenarios—ransomware rumor, outage rumor, and exfiltration scam—and eight seeds per scenario, all shielded policies achieved zero executed protocol violations. Relative to naive coupled control, IGC reduced cyber-harm area under the curve (AUC) by 57.6% in ransomware rumor and 42.6% in outage rumor while also reducing misbelief. Results were scenario-dependent rather than uniformly dominant: in exfiltration scam, a broadcast-only ablation outperformed targeted messaging, showing that targeting can fail when diffusion rapidly crosses community boundaries. Sensitivity analysis further shows that IGC attenuates the brittleness observed under strong coupling and weak moderation. The results suggest that biomimetic regulation is valuable not because coupling always helps, but because it prevents overreaction, clarifies when targeting should be used, and yields safer organizational defaults for misinformation-aware incident response.

## 1. Introduction

Cyber incidents are rarely confined to technical containment. Ransomware disruptions, service outages, suspected data-exfiltration events, and follow-on scam waves now unfold simultaneously across enterprise systems and public information environments. As soon as operational symptoms become visible, customers, employees, and service users begin to interpret what is happening, exchange explanations, and react to both official and unofficial messages. Adversaries exploit the same window through rumor, coordinated disinformation, and opportunistic fraud. The resulting crisis is therefore socio-technical rather than purely technical: cyber conditions shape public behavior, and public behavior in turn alters cyber outcomes. This premise is central to social cybersecurity, which treats human behavior and information flows as integral parts of security performance rather than as external contexts [1,2].

From an incident response perspective, this interdependence matters for concrete operational reasons. Misinformation can suppress reporting of suspicious activity, reduce compliance with mitigation guidance, overload support channels with false leads, and redirect affected users toward phishing or fraud. At the same time, official communication is safety-critical. A premature debunk, unsupported attribution, or over-disclosure of sensitive details can create panic, increase legal or privacy exposure, undermine future trust, and constrain the response team’s options. In organizational crises, communication is therefore not a public-relations afterthought; it is an operational intervention that can either reduce or amplify harm. This operational interpretation is consistent with the social-mediated crisis communication model, which shows that crisis publics actively seek information across official, interpersonal, and social-media channels [3]. Later assessments show that this literature has developed into a substantial global research stream [4]. Trust is also central in crisis information environments, because degraded trust can weaken how misinformation is contested and how official guidance is received [5].

Despite these bidirectional dependencies, the two relevant control loops are usually studied in isolation. Autonomous cyber-defense environments such as CybORG, CybORG++, and CyberBattleSim emphasize containment, restoration, and adversarial uncertainty on the technical side [6,7,8,9], but typically omit public trust, rumor dynamics, and official messaging as a governed action channel. Conversely, misinformation research has produced valuable work on false-news diffusion [10,11], susceptibility to misinformation [12], and intervention design. In particular, prebunking and inoculation studies examine how resistance can be built before exposure [13], platform-intervention work studies how moderation affects fake-news dissemination [14], and recent synthesis work organizes individual-level tools such as prompts and corrections [15]. Adjacent crisis-communication studies show that organizational correction design also matters: internal crisis corrections require strategies tailored to employee communication [16], correction placement and refutation detail alter how publics respond to crisis misinformation [17], and prebunking effectiveness depends in part on spokesperson connection and perceived correction quality [18]. Yet these studies usually evaluate information-layer or communicative outcomes rather than closed-loop effects on detection, compliance, restoration, or cyber harm. Safe-control and shielding research shows that runtime enforcement can guarantee safe executed actions [19,20], but these ideas have not been developed for defender-side crisis communication embedded in a coupled cyber–social incident response loop.

This paper addresses that gap at the *organizational* level. We focus on acute cyber crises in which a defender and an official communications function jointly manage incidents affecting customers, employees, or service users. Our scope is the operational setting faced by security operations centers, incident commanders, and crisis communication teams during ransomware disruptions, ambiguous outages, and exfiltration-related scam waves. We do *not* attempt to model state-level information operations, long-horizon geopolitical influence campaigns, or platform-wide governance optimization. The problem here is narrower and more operational: how to coordinate cyber response and public messaging under uncertainty when public behavior changes the incident itself.

That framing raises three design questions. First, if public sentiment measurably affects reporting and compliance, when should a controller trust cyber–social coupling rather than ignore it or overreact to it? Second, when misinformation risk is heterogeneous across communities, when is targeted correction actually better than broad communication, and when does targeting delay the response too much to help? Third, can communication governance be enforced at runtime so that unsafe messages do not execute even when the controller is operating under pressure and partial information? These questions are practical as well as theoretical because real organizations need both effectiveness and governance: a response policy that improves average outcomes but occasionally emits unsafe public statements is not deployable.

1. **RQ1:** Under what conditions does cyber–social coupling improve incident response outcomes relative to decoupled baselines?
2. **RQ2:** When does targeted counter-messaging outperform broadcast communication, and when does it fail?
3. **RQ3:** Can runtime protocol enforcement eliminate unsafe communications (zero executed violations) while preserving operational effectiveness?

To address these questions, we introduce V-CHIMERA, an immune-inspired verified framework for misinformation-aware organizational incident response. The framework models cyber operations and public communication as coupled control loops over a shared cyber–social state. It combines three main mechanisms. First, an explicit cyber–social coupling architecture links cyber state, belief dynamics, trust, and behavior channels such as compliance and reporting. Second, a runtime communication shield enforces hard protocol constraints on executed messages, separating soft performance objectives from non-negotiable communication safety. Third, an immune-gated coupling (IGC) regulator uses danger signaling, tolerance thresholds, and immune memory to decide when social feedback should influence cyber response and how aggressively counter-messaging should be scaled and targeted.

We study the framework in a coupled cyber–social testbed across three representative organizational crisis scenarios: ransomware rumor, outage rumor, and exfiltration scam. The empirical question is not whether coupling is always beneficial, but whether it can be made selective enough to avoid brittleness. The evaluation shows that shielded policies achieve zero executed protocol violations, immune-gated coupling substantially improves on naive coupled control in ransomware rumor and outage rumor, and broadcast-only communication can outperform targeted messaging in exfiltration scam. The main lesson is therefore conditional rather than universal: coupling can help, but only when it is regulated, evidence-sensitive, and allowed to fall back to broader communication when the crisis becomes population-wide.

We make four contributions:

1. **New problem setting.** We formulate misinformation-aware organizational cyber crisis response as a coupled cyber–social control problem in which operational cyber actions and official public communication jointly act on a shared partially observed state under explicit communication-governance constraints.
2. **Novel method.** We introduce V-CHIMERA, which combines an explicit cyber–social coupling architecture, a runtime communication shield, and an immune-inspired supervisory regulator that gates when social feedback should affect cyber response and how counter-messaging should escalate.
3. **Extensive evaluation.** We evaluate the framework across three crisis scenarios, multiple ablations, exact paired permutation tests, secondary metrics beyond AUC, and a sensitivity analysis over coupling strength and moderation.
4. **Reproducible artifact and deployment guidance.** We provide a scriptable research artifact together with shared defaults, scenario templates, and calibration guidance intended to support both replication and organizational prototyping.

The remainder of the paper is organized as follows. Section 2 provides background on cyber–social feedback, partial observability, and safety-critical communication. Section 3 positions the work relative to misinformation intervention, social cybersecurity, cyber-defense simulation, safe control, and artificial immune systems. Section 4 presents the formal framework and immune-gated controller. Section 5 describes the experimental setup. Section 6 reports the main results, robustness checks, and temporal dynamics. Section 7 discusses deployment guidance and limitations, and Section 8 concludes.

## 2. Background

The formal framework developed in Section 4 rests on the premise that organizational cyber crises are not purely technical events. They are coupled cyber–social processes in which operational conditions, public narratives, user behavior, and official communication evolve together. This premise has three important consequences. First, incident response must account for both cyber-state and social-state dynamics rather than treating communication as an external afterthought. Second, the relevant cyber and social variables are only partially observed, so controllers must act on noisy and delayed evidence rather than on ground truth. Third, public communication is a safety-critical intervention: some messages may be harmful enough that they should be disallowed outright rather than merely penalized in expectation. These considerations motivate the later design of V-CHIMERA as a coupled, constrained control architecture rather than as either a cyber-only defender or an isolated misinformation intervention model.

### 2.1. Cyber–Social Feedback in Organizational Crises

A cyber crisis unfolds in a human–information–machine loop. Technical events such as service outages, ransomware activity, or suspected exfiltration create narrative shocks: users seek explanations, adversaries exploit ambiguity, and online discussion can rapidly amplify speculation, rumor, or opportunistic fraud. This pattern is consistent with the social-mediated crisis communication model, which treats crisis publics as active information seekers moving across official, interpersonal, and social-media channels rather than relying on a single source [3]. Those narratives do not remain purely discursive. They affect whether users believe official guidance, whether they comply with requests to patch, isolate devices, or ignore scams, and whether they report suspicious activity quickly enough for defenders to detect and contain the incident.

For this reason, the cyber and social layers should be understood as reciprocally coupled. Cyber conditions influence the social layer by changing what audiences infer about the organization’s competence, honesty, and level of control. The social layer influences the cyber layer by changing two behavior channels that matter operationally: *compliance* with recommended actions and *reporting* of suspicious or relevant observations. When compliance falls, even technically sound containment guidance may fail to take effect at the population level. When reporting falls, detection and triage degrade because defenders receive fewer timely signals from affected users. Conversely, when public communication successfully reduces confusion and increases trust, reporting and cooperation can improve, thereby changing incident evolution rather than merely changing perception. Recent work on crisis-information trust further reinforces this point: degraded trust in the surrounding social-media information environment can weaken the credibility of corrective communication precisely when organizations most need rapid reporting and compliance [5].

Classical opinion-dynamics models such as DeGroot consensus and bounded-confidence systems provide useful abstractions for belief evolution under social influence [21,22]. However, organizational crises differ from stylized consensus settings in several important ways. Influence is heterogeneous across communities, communication unfolds across multiple platforms rather than one homogeneous network, and the information environment may be adversarially manipulated through bots, coordinated narratives, or follow-on scams. In addition, official communication is not just another peer-to-peer signal. It is an intervention channel with different epistemic weight, different timing, and different governance constraints. These features make an agent-based, multi-platform treatment more appropriate than a single aggregate-population model.

This framing also changes the interpretation of crisis communication. In many organizational settings, communication is treated as post hoc explanation of operational events that have already been decided elsewhere. In the coupled setting used here, communication is better understood as an *operational control surface*. It can reduce incident harm by improving compliance, increasing reporting, narrowing uncertainty, and limiting follow-on victimization; it can also amplify harm if it is mistimed, overconfident, or poorly targeted. The later framework therefore treats messaging as a co-equal control channel rather than as a passive output of the cyber response process.

### 2.2. Coupled Decision-Making Under Partial Observability

The need for coupled control is inseparable from the problem of partial observability. Neither cyber state nor social state is directly known during a live crisis. On the cyber side, telemetry may be incomplete, delayed, or ambiguous, especially in the early stages of an incident when service degradation is visible but root cause remains uncertain. On the social side, the organization does not observe “true” belief, trust, or uncertainty directly. Instead, it sees proxies such as help-desk volume, phishing reports, engagement with official announcements, sampled posts, surveys, or sentiment summaries, all of which are noisy, lagged, and vulnerable to manipulation.

This has two implications for control design. First, cyber response and public communication cannot be optimized independently, because each controller acts on a partial view of a shared evolving environment. A communication action changes not only social metrics but also future cyber observations through reporting and compliance. A cyber action changes not only operational state but also the future information environment by creating or resolving ambiguity. Second, raw social signals should not be trusted unconditionally. A spike in sentiment may reflect genuine confusion, but it may also reflect coordinated amplification, temporary rumor cascades, or sampling artifacts. A well-designed controller must therefore distinguish between *informative* social feedback and *misleading* social noise.

These considerations motivate the later use of a shared belief and sentiment estimate that conditions both controllers. The estimator is not meant to recover an exact latent state; its purpose is more modest and more operationally relevant: to summarize available cyber and social evidence into a coherent working state that supports coordinated action. In this sense, V-CHIMERA treats the crisis as a coupled decision problem under uncertainty rather than as a static communication task or a purely technical incident simulator.

The same reasoning identifies compliance and reporting as central coupling variables. They are not merely attitudes; they are the most direct mechanisms by which public behavior changes operational effectiveness. If social feedback is routed into the cyber controller at all, it should be routed through behavior channels that have a clear operational interpretation rather than through sentiment in the abstract. This helps keep the coupling map interpretable and makes later calibration more plausible in real organizational settings.

### 2.3. Safety-Critical Communication and Biomimetic Regulation

A second conceptual pillar of the framework is the distinction between *soft* performance objectives and *hard* communication safety. Runtime shielding provides a clean way to enforce that distinction: a controller may optimize over ordinary objectives, but a shield restricts what may actually execute by enforcing explicit state-dependent constraints [19,20]. This is especially appropriate for crisis communication. A single unsafe message—for example, a premature debunk, an unsupported attribution, or an instruction that exceeds the available evidence—can produce irreversible legal, operational, or reputational harm even if average performance improves. For such cases, treating safety only as a penalty term is inadequate. Hard runtime governance is conceptually more faithful to how real organizations manage public crisis communication. Crisis-correction studies also show that intervention design matters: internal debunking requires communication strategies suited to the affected organizational audience [16], correction placement and refutation detail affect how publics respond to misinformation narratives [17], and prebunking quality can influence communicative response [18].

The third pillar is biomimetic regulation. Biological immune systems offer a useful control metaphor because they must make escalation decisions under uncertainty while avoiding both underreaction and harmful overreaction. Danger theory emphasizes that response should be triggered by credible signals of harm rather than by pattern matching alone [23]. Artificial immune systems extend related ideas—danger signals, immune memory, tolerance thresholds, and graded response—to computational settings involving noisy evidence and adaptive regulation [24,25]. These ideas are relevant here not because organizational crisis response literally reproduces immune physiology, but because they provide a disciplined vocabulary for deciding when coupling should activate, when targeting should persist, and when escalation should be damped.

Four aspects of the immune analogy are particularly useful for the present problem. First, *danger signaling* motivates the idea that coupling should depend on credible cyber harm and evidential state rather than on social activation alone. Second, *tolerance* motivates withholding reliance on social feedback when uncertainty is too high or when signals are insufficiently trustworthy. Third, *immune memory* motivates smoothing community-level risk over time so that targeting responds to persistent misinformation pressure rather than one-step spikes. Fourth, *checkpointing* motivates the runtime shield as a final safety layer that prevents harmful public actions from executing even when the upstream controller is highly activated.

Taken together, these background concepts motivate the architecture. The framework is coupled because cyber crises are behaviorally mediated; it is partially observed because both operational and social evidence are incomplete; it is verified because communication is safety-critical; and it is immune-inspired because the central control problem is not merely how to react, but how to regulate reaction under uncertainty.

## 3. Related Work

V-CHIMERA sits at the intersection of eight research streams: social cybersecurity, social-mediated crisis communication, organizational corrective communication, misinformation intervention, autonomous cyber-defense simulation, multi-agent coordination and security decision-making, safe constrained control, and biomimetic regulation. Each stream contributes an important part of the present problem, but none addresses the full organizational setting studied here: a defender and an official communications function acting in a closed loop over a coupled cyber–social state under hard runtime communication constraints. Table 1 summarizes representative studies and makes the gap more explicit.

Research in *social cybersecurity* establishes the central premise that cyber outcomes depend not only on technical compromise, but also on human behavior, influence operations, and online information flows [1,2]. This literature is essential for motivating why trust, reporting, and narrative dynamics belong inside cyber defense at all. However, most work in this area is diagnostic, conceptual, or survey-oriented: it characterizes the threat surface and documents the importance of human and network effects, but it does not specify a closed-loop controller that jointly allocates operational cyber actions and official crisis communication within a live incident.

A second line of work examines social-mediated crisis communication and organizational corrective communication. The social-mediated crisis communication model explains how publics seek crisis information across official, interpersonal, and social channels during crises [3], and updated assessments show that this literature has developed into a substantial global research stream [4]. Recent crisis-misinformation studies extend this line by focusing on trust and corrective design: trust in social-media information environments affects how misinformation is contested during crises [5]; internal crisis-communication research studies how organizations should debunk misinformation among employees [16]; correction placement and refutation detail shape public responses to crisis misinformation [17]; and prebunking quality influences publics’ communicative responses [18]. This stream is the closest communication-side literature to the present setting, but its outcomes remain communicative and perceptual rather than operational cyber outcomes such as detection, containment, reporting, or incident harm.

A third line of work studies *misinformation interventions*. Foundational diffusion studies show that false information spreads in distinctive ways online [10,11], while susceptibility research shows that reasoning and attention shape vulnerability to false content [12]. Intervention studies then examine prebunking and inoculation [13], platform moderation [14], and individual-level tools such as prompts and corrections [15]. Taken together, these studies show that well-designed interventions can reduce susceptibility, alter diffusion, and improve information quality. Yet their outcomes are typically information-layer outcomes, such as belief accuracy, sharing, or exposure, rather than operational cyber outcomes such as detection, restoration, reporting, or incident harm. The missing step for the present paper is therefore not another intervention in isolation, but the closure of the loop from public communication back into operational cyber performance.

A fourth literature concerns *autonomous cyber-defense simulation*. Environments such as CybORG, CybORG++, and CyberBattleSim provide valuable benchmarks for defender policies under uncertainty and adversarial progression [6,7,8,9]. These platforms are important because they make cyber decision-making measurable and reproducible. However, they generally treat the public information environment as exogenous. They do not model misbelief, trust erosion, crisis communication, community targeting, or the possibility that public behavior changes cyber outcomes through compliance and reporting. As a result, they cannot evaluate the type of cyber–social trade-off that is central to this paper.

A fifth stream provides broader foundations for multi-agent coordination and security decision-making. Multi-agent systems research formalizes coordination among interacting decision-makers [26,27], work on machine behavior highlights that algorithmic agents can create collective dynamics that require social-level analysis [28], and security game theory develops strategic models for allocating defensive resources under adversarial pressure [29]. These literatures motivate the coordinated decision-making perspective used here, but they do not address misinformation-aware organizational crisis communication, runtime message governance, or bidirectional coupling between public behavior and cyber incident evolution.

A sixth line addresses *safe constrained control and runtime shielding*. Shielding work shows that hard runtime constraints can prevent unsafe executed actions even when the underlying policy is imperfect [19,20]. This idea is directly relevant here because public crisis communication is safety-critical: a single unsupported or premature message can create irreversible operational, legal, or reputational harm. However, existing shielding work is largely developed in the context of reinforcement learning and generic safe control. It does not address defender-side organizational messaging under misinformation-aware incident response, nor does it address the distinct question of when social feedback should influence cyber operations in the first place.

Finally, *artificial immune systems* and related biomimetic control approaches provide the conceptual lineage for the immune-gated component of V-CHIMERA [24,25]. These works contribute useful ideas such as danger signaling, immune memory, tolerance thresholds, and graded response under uncertainty. Their value for the present paper is structural rather than domain-specific: they motivate a regulator that escalates only under credible danger, smooths transient risk, and avoids overreaction. But prior immune-inspired systems are not formulated as verified controllers for coupled organizational cyber–social incident response.

Two observations follow from this comparison. First, the closest prior works each omit at least one component that is essential in the present setting: a coupled cyber–social state, defender-side crisis communication as a co-equal control channel, hard runtime governance for public messaging, or adaptive regulation of when social feedback should influence cyber operations. Second, there is no directly comparable benchmark in which all of these components coexist. For that reason, the comparison in this paper is necessarily architectural and methodological rather than a one-to-one numerical benchmark against a single prior system.

Relative to this literature, the novelty of V-CHIMERA is therefore not that each ingredient is individually new. Rather, the contribution is the *verified integration* of four elements, with runtime verification scoped to the communication-shield layer, that are usually studied separately: (i) coupled cyber and social state dynamics, (ii) defender-side crisis communication as a co-equal control channel, (iii) hard runtime protocol enforcement for public messaging, and (iv) an immune-inspired supervisory regulator that decides when social feedback should affect cyber response and how counter-messaging should be scaled. Compared with cyber-only simulators [6,7,8,9], we add public trust, misbelief, and communication governance. Compared with social-mediated crisis communication and organizational corrective-communication work [3,4,5,16,17,18], we add explicit cyber-operational feedback, behavior channels such as reporting and compliance, and runtime governance. Compared with misinformation-intervention studies [10,11,12,13,14,15], we close the loop back into operational cyber outcomes. Compared with safe reinforcement learning shielding work [19,20], we apply hard runtime safety to organizational crisis communication. Compared with artificial immune-system work [24,25], we operationalize biomimetic regulation inside a shielded cyber–social incident-response architecture.

## 4. V-CHIMERA Framework and Methodology

### 4.1. Design Objectives and Formal Problem Setting

We adopt a design-science perspective [30,31], but the artifact is not merely descriptive. It is a prescriptive control architecture for organizational cyber crises in which technical response and official communication interact through a shared cyber–social environment. The central design problem is therefore not only how to optimize incident response, but how to do so when public behavior feeds back into operational outcomes and when communication itself is subject to non-negotiable governance constraints.

Let

$$s_{t}=\left(s_{t}^{C},s_{t}^{S}\right)$$

denote the joint cyber–social state at time *t*, where $s_{t}^{C}$ contains cyber variables such as compromise status, service availability, and exfiltration risk, and $s_{t}^{S}$ contains social variables such as misbelief, trust, uncertainty, and polarization. Two coordinated decision-makers act at each step: a cyber controller with action $a_{t}^{C}\in A^{C}$ and a communications controller with action $a_{t}^{M}\in A^{M}$. Because the state is only partially observed, the controllers do not act on $s_{t}$ directly. Instead they maintain a shared belief estimate

$$b_{t}\approx p\left(s_{t}|o_{\leq t},a_{<t}^{C},{\tilde{a}}_{<t}^{M}\right),$$

where $o_{t}=\left(o_{t}^{C},o_{t}^{S}\right)$ are the current cyber and social observations and ${\tilde{a}}_{t}^{M}$ denotes the message that actually executes after runtime enforcement.

The coupled environment can be written at a high level as two interacting transition processes:

(1) $$s_{t+1}^{C}∼P^{C}\left(\;\cdot |s_{t}^{C},a_{t}^{C},\phi_{t}^{SC}\right),\;\phi_{t}^{SC}=\Phi_{SC}\left(s_{t}^{S}\right),$$

(2) $$s_{t+1}^{S}∼P^{S}\left(\;\cdot |s_{t}^{S},{\tilde{a}}_{t}^{M},\phi_{t}^{CS}\right),\;\phi_{t}^{CS}=\Phi_{CS}\left(s_{t}^{C},a_{t}^{C}\right).$$

Here Φ*_SC_* is the social-to-cyber coupling map and Φ*_CS_* is the cyber-to-social shock map. This factorization makes the main modeling commitment explicit: cyber and social dynamics are not collapsed into one opaque transition kernel, but are coupled through interpretable cross-domain channels.

Over a finite horizon *T*, the soft control objective is to reduce cyber harm and misinformation while preserving trust:

(3) $$R_{t}=-\alpha H\left(s_{t}^{C}\right)-\beta M\left(s_{t}^{S}\right)+\gamma T\left(s_{t}^{S}\right),$$

and

$$J\left(\pi \right)=E_{\pi }\;\left[\sum_{t=0}^{T-1}R_{t}\right].$$

Here *H* is cyber harm, *M* is misbelief, and T is trust. This notation keeps the trust term distinct from the finite horizon *T*. Uncertainty and polarization are not placed directly in the reward in the present implementation; instead, they enter the controller through the immune gate and are evaluated explicitly in the experiments.

Communication safety is treated differently. Unsafe messages are not merely penalized in the reward; they are excluded from the executed action set. This separation is deliberate. In organizational crises, a rare but severe communication failure can be unacceptable even if average performance is good. We therefore treat communication governance as a hard state-dependent feasibility condition:

$$A_{\mathrm{safe}}^{M}\left(s_{t}\right)=\left\{a\in A^{M}:g_{k}\left(s_{t},a\right)\leq 0\;\mathrm{for}\;\mathrm{all}\;k\right\},$$

and require that the executed message satisfy ${\tilde{a}}_{t}^{M}\in A_{\mathrm{safe}}^{M}\left(s_{t}\right)$ at every step.

This formulation encodes four design requirements that motivate the rest of the framework. First, *safety separability*: performance may be optimized under soft objectives, but executed communications must remain protocol-safe by construction. Second, *evidential conservatism*: social-to-cyber coupling should not activate when operational danger is weak or social signals are too uncertain to trust. Third, *persistence-sensitive targeting*: local intervention should respond to sustained rather than one-step misinformation spikes. Fourth, *regime adaptivity*: when a crisis becomes population-wide, the controller should revert from narrow targeting to broader messaging.

### 4.2. Architecture and Coupling Bus

**Figure 1:**
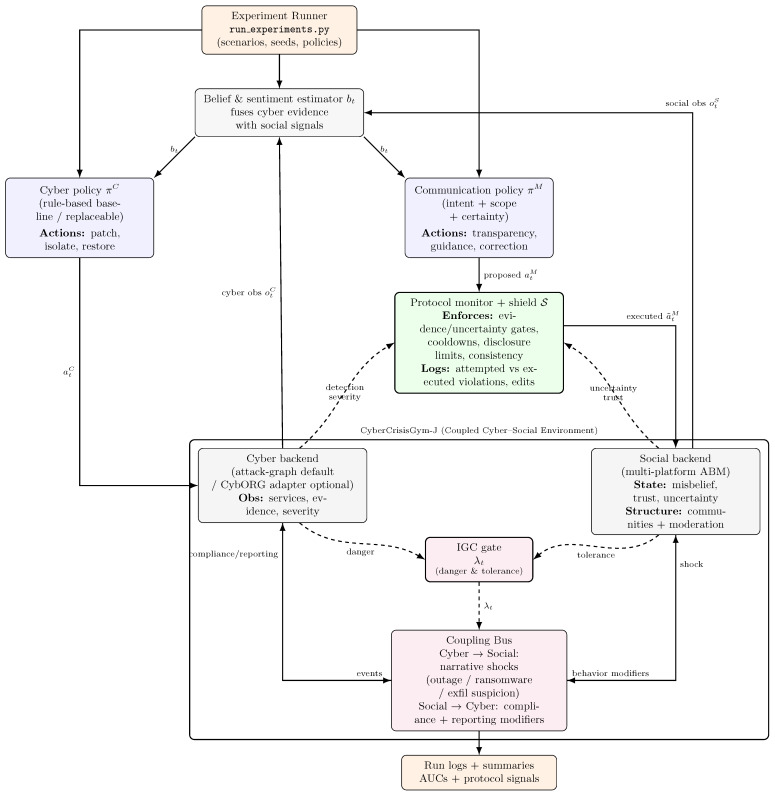
V-CHIMERA architecture. Two cooperating agents select cyber operations and public communications. A belief and sentiment estimator *b_t_* aggregates cyber and social observations and conditions both policies. Communication actions are filtered by a runtime protocol shield that guarantees zero executed protocol violations while logging attempted violations and interventions. A coupled cyber–social environment (CyberCrisisGym-J) models bidirectional feedback between operational state and social dynamics, while an IGC gate computes *λ_t_* from cyber danger and social tolerance to modulate cross-domain coupling. Colors group functional modules (estimation, controllers, shielding, environment, coupling, and logs) for readability rather than indicating quantitative values. Experiment logs are aggregated into the reported tables and figures.

Figure 1 instantiates the above formulation as two coordinated control loops connected by a shared estimator and a bidirectional coupling bus. The shared belief and sentiment estimator compresses noisy cyber and social observations into a working posterior *b_t_* that conditions both policies. The cyber controller chooses operational actions such as patching, restoration, isolation, and monitoring. The communications controller chooses a structured message proposal consisting of an intent, a target scope, an evidence tag, an uncertainty label, and an intensity. The runtime shield then decides what may actually execute.

The coupling bus provides the only cross-domain pathways. In the social-to-cyber direction, the environment passes two behavior channels to the cyber backend. Let ${\mathrm{comp}}_{t}\in \left[0,1\right]$ denote the population-average compliance propensity at time *t*, that is, the expected fraction of affected agents who follow current organizational guidance such as patching, credential reset, isolation, or scam avoidance. Let ${\mathrm{rep}}_{t}\in \left[0,1\right]$ denote the population-average reporting propensity, that is, the expected fraction of agents who submit a report or help-desk signal after observing suspicious activity. These channels are derived from the underlying agent-level social state and then summarized at the population level before being passed to the cyber backend. A value of 0.5 is treated as behaviorally neutral: values above 0.5 strengthen defender effectiveness, whereas values below 0.5 weaken detection and mitigation.

The implemented social-to-cyber map uses linear modifiers around this neutral baseline:

(4) $${\mathrm{comp}}_{t}^{\prime }=0.5+s_{SC}\left({\mathrm{comp}}_{t}-0.5\right),\;{\mathrm{rep}}_{t}^{\prime }=0.5+s_{SC}\left({\mathrm{rep}}_{t}-0.5\right),$$

where *s_SC_* is the scenario-specific social-to-cyber coupling scale. When *s_SC_* = 1, the social signal is passed through unchanged; when 0 < *s_SC_* < 1, deviations from neutral are damped; and when *s_SC_* > 1, they are amplified. Equation (4) has two useful design properties. It is *neutrality preserving*, because the baseline value 0.5 is a fixed point, and it is *direction preserving*, because stronger compliance or reporting can only improve the modifier when *s_SC_* > 0, while weaker compliance or reporting can only degrade it.

In the opposite direction, cyber conditions generate narrative shocks into the social layer. At a high level, this can be written as

$$\xi_{t}=W_{CS}z_{t}^{C},$$

where $z_{t}^{C}$ stacks normalized cyber indicators such as service disruption, ransomware confirmation, incident severity, and exfiltration risk, and *W_CS_* maps them into social perturbations. The social backend then uses $\xi_{t}$ to update misbelief, trust, and uncertainty. This abstraction is important conceptually: cyber events do not influence the social model as generic “bad news,” but through structured narrative shocks whose weights can differ by scenario.

### 4.3. Immune-Gated Coupling as Supervisory Regulation

Naive coupling is brittle because social signals early in an incident may be sparse, delayed, noisy, or adversarially manipulated. A controller that reacts too directly to such signals can amplify disruption, trigger premature correction, or overfit to transient local spikes. We therefore cast immune-gated coupling (IGC) as a *supervisory regulator* over an otherwise ordinary coupled controller. The regulator does not replace cyber or messaging policies; it governs when cross-domain coupling is trusted, how targeting is selected, and how strongly communication escalates.

The first supervisory signal is a scalar danger score:

(5) $$d_{t}=\mathrm{clip}\;\left(w_{\mathrm{sev}}\;{\mathrm{sev}}_{t}+w_{\mathrm{gap}}\max\left(0,\theta_{\det}-{\mathrm{conf}}_{t}\right),\;0,\;1\right),$$

where sev*_t_* is normalized cyber severity, conf*_t_* is detection confidence, *θ*_det_ is the “good enough” verification reference, and $w_{\mathrm{sev}}+w_{\mathrm{gap}}$ determine how strongly the regulator responds to realized disruption versus lack of verification. This construction makes the intended logic explicit. Severe incidents can raise danger even under good evidence, while weak evidence raises danger only when confidence remains below the verification reference. The controller therefore treats unresolved ambiguity as a reason for caution, not as a reason for confident escalation.

Coupling is activated only when operational danger is sufficiently high and social measurements are informative enough to trust:

(6) $$\lambda_{t}=I\;\left[d_{t}\geq \tau_{d}\wedge u_{t}<\tau_{u}\right],$$

where $u_{t}$ is aggregate social uncertainty. The binary variable $\lambda_{t}$ is a supervisory switch, not a reward term. When $\lambda_{t}=0$, the cyber controller falls back to a conservative decoupled rule set; when $\lambda_{t}=1$, the controller is allowed to use the social-to-cyber modifiers from Equation (4). This realizes a tolerance-style principle: potentially informative social feedback is not ignored forever, but it is withheld from operational control until both danger and informativeness conditions are met.

Targeting is then driven by persistent community-level risk rather than by instantaneous sentiment alone. For each community *c*, we compute an antigen score

(7) $$a_{c,t}=w_{M}m_{c,t}+w_{T}\left(1-r_{c,t}\right)+w_{U}u_{t},$$

where $m_{c,t}$ and $r_{c,t}$ are community-level misbelief and trust, and $u_{t}$ is aggregate uncertainty. At the aggregate level, we reserve $m_{t}$ for overall misbelief and $r_{t}$ for overall trust so these variables are not confused with the shared belief estimate $b_{t}$. The regulator then maintains an immune-memory state

(8) $$q_{c,t}=\delta q_{c,t-1}+\left(1-\delta \right)a_{c,t},$$

which smooths transient spikes and stabilizes the ranking of at-risk communities.

This memory term is theoretically important. Without it, targeting would react to one-step fluctuations in local sentiment, which is precisely the kind of brittleness the regulator is meant to suppress. With *δ* close to one, a community must exhibit sustained antigen load before it becomes a stable targeting candidate.

Define the eligible target set

$$C_{t}=\left\{c:q_{c,t}\geq \tau_{a}\right\}.$$

If targeting is enabled and *C_t_* ≠ ∅, the controller chooses $c^{*}=\arg\max_{c\in C_{t}}q_{c,t}$; otherwise it broadcasts. Even when an eligible community exists, the implemented controller falls back to all-audience communication when global misbelief exceeds $\theta_{M}$ or trust falls below $\theta_{T}$, because elevated macro misbelief or fragile trust is evidence that the crisis has become broadly salient and that narrow segmentation may no longer be appropriate.

Message intensity follows a clonal-expansion logic:

(9) $$I_{t}=\mathrm{clip}\left(I_{0}+\kappa q_{c^{*},t},\;0.15,\;0.90\right)\times \mathrm{clip}\left(0.40+0.60r_{t},\;0.40,\;1.00\right),$$

where $c^{*}$ is the selected community (if any) and $r_{t}$ is aggregate trust. The first factor increases response strength with persistent antigen load. The second factor damps intensity when trust is already low, reducing the risk of backfire from highly assertive messaging in distrustful regimes.

The resulting regulator satisfies four useful monotonicity and robustness properties. Increasing severity or decreasing confidence weakly increases $d_{t}$; increasing community misbelief or decreasing community trust weakly increases $a_{c,t}$; transient spikes affect $a_{c,t}$ immediately but only persistent spikes drive $q_{c,t}$ above $\tau_{a}$; and once misbelief becomes broadly elevated or trust becomes fragile, the policy abandons narrow targeting in favor of broadcast. These are the theoretical reasons the immune analogy is useful here. The value of the analogy is not biological realism, but a disciplined vocabulary for escalation, tolerance, persistence, and checkpointing under uncertainty.

Table 2 summarizes this mapping. The immune analogy is functional rather than biologically literal: it is intended to clarify the control logic, not to claim that organizational crisis response literally behaves like immune physiology.

### 4.4. Communication Protocol and Runtime Shield

The communications controller proposes a structured message action

$$a_{t}^{M}=\left(\iota_{t},\omega_{t},e_{t},l_{t},I_{t}\right),$$

where $\iota_{t}$ is the intent (e.g., silence, transparency update, debunk, prebunk, or request for reports), $\omega_{t}$ is the target scope, $e_{t}$ is the evidence flag, $l_{t}$ is the uncertainty label, and $I_{t}$ is the intensity. A proposal is safe only if all protocol predicates are satisfied:

(10) $$\forall k,\;g_{k}\left(s_{t},a_{t}^{M}\right)\leq 0.$$

In the artifact, the main predicates enforce (i) a minimum cooldown between public messages, (ii) evidence requirements for debunks, (iii) explicit uncertainty labeling when uncertainty is high, and (iv) a severity gate that prevents debunking during high-severity, weak-evidence phases.

Abstractly, the shield can be written as the projection

(11) $${\tilde{a}}_{t}^{M}=\arg\min_{a\in A_{\mathrm{safe}}^{M}\left(s_{t}\right)}d_{E}\left(a,a_{t}^{M}\right),$$

where $d_{E}$ is a discrete edit distance over intent, target scope, evidence tag, uncertainty label, and intensity. In other words, the shield executes the proposal unchanged if it is already safe; otherwise it returns the nearest compliant alternative. In the present implementation, the nearest alternative is typically a downgrade to a transparency update, a request for reports, or silence. The shield never fabricates evidence, never upgrades confidence, and always logs its intervention.

**Lemma** **1** ((shielded protocol safety)). *Assume that, for every reachable protocol state s_t_, the safe communication set $A_{\mathrm{safe}}^{M}\left(s_{t}\right)$ is non-empty and that the runtime shield in Equation (11) returns an action ${\tilde{a}}_{t}^{M}\in A_{\mathrm{safe}}^{M}\left(s_{t}\right)$. Then every executed communication action satisfies all protocol predicates in Equation (10); equivalently, the episode has zero executed protocol violations with respect to the encoded predicates.*

**Proof.**  At any time *t*, the message proposed by the communications controller is either already in $A_{\mathrm{safe}}^{M}\left(s_{t}\right)$ or is replaced by the shield with an element of that set. By definition of $A_{\mathrm{safe}}^{M}\left(s_{t}\right)$, every action in the set satisfies $g_{k}\left(s_{t},a\right)\leq 0$ for all predicates *k*. Hence the executed action ${\tilde{a}}_{t}^{M}$ satisfies all protocol predicates at time *t*. Applying the same argument independently at each step of an episode gives zero executed violations over the full rollout. In the implementation, the non-emptiness condition is satisfied by including compliant fallback actions such as silence or downgraded transparency updates.    □

This operator gives the framework a clean safety interpretation. Reward design and control logic determine what the policy would like to say; the shield determines what may actually be said. A penalty term inside the reward could make unsafe communication costly on average, but it could not guarantee the absence of rare catastrophic violations. Lemma 1 states the corresponding construction-level guarantee, while the attempted-violation and edit logs retain auditability for proposals that were corrected before execution.

### 4.5. Algorithms

Table 3 defines the symbols and shared defaults used in Equations (5)–(9). These defaults are shared across all journal-main scenarios; only the environment and coupling YAML files change by scenario. Algorithms 1 and 2 then instantiate the supervisory logic. Algorithm 1 computes danger, updates immune memory, selects message scope and intensity, and proposes a cyber–communication action pair. Algorithm 2 composes the estimator, coupling bus, controller, shield, and environment into one closed-loop episode rollout.

Algorithms 1 and 2 summarize the per-step immune-gated controller and the closed-loop episode rollout, respectively. In the pseudocode, $m_{t}$ denotes aggregate misbelief, $r_{t}$ aggregate trust, and $b_{t}$ remains reserved for the shared belief-state estimate used elsewhere in the framework.
**Algorithm 1** Immune-gated coupling controller at time *t***Require:** observation $o_{t}$ with cyber and social features, previous immune memory ${\left\{q_{c,t-1}\right\}}_{c}$, controller configuration Θ**Ensure:** cyber action $a_{t}^{C}$, proposed communication action $a_{t}^{M}$, updated memory ${\left\{q_{c,t}\right\}}_{c}$, coupling flag $\lambda_{t}$  1:extract ${\mathrm{sev}}_{t}$, ${\mathrm{conf}}_{t}$, evidence availability, aggregate misbelief $m_{t}$, aggregate trust $r_{t}$, uncertainty $u_{t}$, aggregate compliance propensity ${\mathrm{comp}}_{t}$, aggregate reporting propensity ${\mathrm{rep}}_{t}$, and community-level sentiment arrays ${\left\{m_{c,t},r_{c,t}\right\}}_{c}$ from $o_{t}$  2:compute danger score $d_{t}$ using Equation (5)  3:set $\lambda_{t}\leftarrow I\left[d_{t}\geq \tau_{d}\wedge u_{t}<\tau_{u}\right]$  4:compute community antigen values $a_{c,t}$ using Equation (7)  5:update immune memory $q_{c,t}$ using Equation (8) for each community *c*  6:**if** targeting enabled and $\max_{c}q_{c,t}\geq \tau_{a}$ **then**  7:      choose target community $c^{*}=\arg\max_{c}q_{c,t}$  8:**else**  9:      set target to all communities10:**end if**11:**if** $m_{t}\geq \theta_{M}$ **or** $r_{t}<\theta_{T}$ **then**12:      override target and broadcast to all communities13:**end if**14:compute message intensity $I_{t}$ using Equation (9)15:**if** $\lambda_{t}=1$ **then**16:      choose cyber action using the coupled rule set17:**else**18:      choose cyber action using the conservative decoupled fallback19:**end if**20:**if** $d_{t}\geq \tau_{d}$ and evidence unavailable **then**21:      propose a transparency update with an uncertainty label22:**else if** evidence available, $m_{t}\geq \theta_{M}$, and ${\mathrm{conf}}_{t}\geq \theta_{\det}$ **then**23:      propose an evidence-based debunk24:**else if** t≤6 and $m_{t}\geq \theta_{M}$ and $r_{t}\geq \theta_{T}$ **then**25:      propose a prebunk26:**else if** ${\mathrm{rep}}_{t}<0.5$ **then**27:      propose a request-for-reports message28:**else**29:      propose silence30:**end if**31:**return** $a_{t}^{C},a_{t}^{M},{\left\{q_{c,t}\right\}}_{c},\lambda_{t}$

**Algorithm 2** V-CHIMERA closed-loop episode rollout
**Require:** environment E, estimator B, cyber/comms controller pair, protocol shield S, horizon *T***Ensure:** episode summary metrics and optional step-level logs  1:initialize $s_{0}$, $\left(o_{0}^{C},o_{0}^{S}\right)$, belief state $b_{0}$, immune memory, and protocol state (e.g., last message time)  2:**for** t=0,…,T−1 **do**  3:      observe $\left(o_{t}^{C},o_{t}^{S}\right)$ and update belief state $b_{t}\leftarrow B\left(b_{t-1},o_{t}\right)$  4:      compute social-to-cyber modifiers $\left({\mathrm{comp}}_{t}^{\prime },{\mathrm{rep}}_{t}^{\prime }\right)$ using Equation (4)  5:      call the policy (Algorithm 1 for IGC, baseline variant otherwise) to obtain proposed $a_{t}^{C}$ and $a_{t}^{M}$  6:      execute the communication shield: ${\tilde{a}}_{t}^{M}\leftarrow S\left(a_{t}^{M},s_{t}\right)$  7:      step the environment with $\left(a_{t}^{C},{\tilde{a}}_{t}^{M}\right)$ and receive $\left(s_{t+1},o_{t+1}\right)$  8:      log cyber harm, misbelief, trust, uncertainty, polarization, attempted violations, executed violations, and shield edits  9:
**end for**
10:aggregate step-level traces into AUC and final-state metrics11:**return** run summaries and optional step logs


## 5. Experimental Setup

The evaluation design addresses the three research questions by separating three issues: whether cyber–social coupling helps at all, whether immune gating stabilizes coupling when social signals are noisy or adversarially shaped, and whether hard runtime enforcement preserves communication safety without collapsing operational usefulness. To do so, we evaluate a fixed set of policies under shared controller defaults, vary only the scenario environments rather than retuning the controller per scenario, and report both effectiveness and governance metrics from the same run logs.

### 5.1. Testbed and Scenarios

We evaluate V-CHIMERA in CyberCrisisGym-J, a coupled cyber–social simulation environment that joins a stochastic cyber incident backend to a multi-platform social agent-based model. The cyber backend tracks compromise progression, service disruption, exfiltration risk, and defender actions such as monitoring, patching, restoration, and hunting. The social backend tracks misbelief, trust, uncertainty, polarization, community structure, bot activity, and platform moderation. The coupled environment therefore supports the two directions of influence that motivate this paper: cyber conditions generate narrative shocks into the social layer, and social state feeds back into cyber operations through aggregate compliance and reporting modifiers.

This design supports interpretation. In many cyber-defense environments, the social layer is absent, so public behavior cannot affect detection or mitigation. In many misinformation studies, the cyber layer is absent, so communication interventions do not feed back into operational incident response. CyberCrisisGym-J is used here precisely because it supports both directions in one closed loop. The purpose of the testbed is not to claim perfect realism for any single organization, but to provide a controlled environment in which coupling, targeting, and runtime governance can be evaluated under identical conditions. Appendix A describes an artifact extension showing how the same coupling and shielding interface can be attached to a CybORG enterprise-defense backend.

The default scenario pack in the artifact is the same one used in this paper: ransomware_rumor, outage_rumor, and exfiltration_scam. These three scenarios were chosen to represent qualitatively different cyber–social operating regimes rather than to exhaust the space of possible incidents.

The ransomware_rumor scenario emphasizes severe operational disruption combined with rumor amplification. It is intended to test whether the controller can coordinate containment and communication when the incident is visibly harmful and misinformation can intensify public reaction during restoration. The outage_rumor scenario is less about confirmed compromise and more about ambiguity: users observe service failure, but the cause is initially unclear, so uncertainty and trust erosion become central. This regime tests whether the controller can remain conservative while evidence is still incomplete. The exfiltration_scam scenario emphasizes follow-on fraud risk and cross-community diffusion. Here the key operational issue is that broad public reporting and warning behavior can matter as much as targeted correction, making the scenario an especially informative test for RQ2.

Each episode lasts 60 time steps. This horizon is long enough to capture early ambiguity, mid-episode escalation, and late-stage stabilization or trust repair under the scenario dynamics used here. All AUC and final-state metrics reported later are computed from the complete 60-step episode traces.

### 5.2. Policies

We compare seven policies. The policy set is structured rather than exhaustive: each policy either serves as a baseline, adds one major mechanism, or removes one major mechanism from the full design. This structure yields controlled comparisons rather than a leaderboard over unrelated methods.

- **Pipeline**: a sequential reference policy consisting of cyber response plus generic communications, without cyber–social coupling and without runtime shielding.
- **Pipeline + Shield**: the same sequential baseline with the runtime communication shield enabled. This isolates the effect of protocol enforcement without changing the baseline response logic.
- **V-CHIMERA**: a naive coupled controller without the shield. This variant allows cyber and communication decisions to react to the shared cyber–social state, but does so without immune gating and without protocol enforcement.
- **V-CHIMERA + Shield**: the same naive coupled controller with the shield enabled. This isolates the effect of protocol enforcement under coupling while still leaving the coupling logic itself unregulated.
- **V-CHIMERA + IGC + Shield**: the full proposed method. This variant adds immune-gated coupling to regulate when social feedback is trusted and how strongly counter-messaging escalates, while preserving hard communication safety through the shield.
- **No coupling + Shield**: an ablation that disables the operational use of the social-to-cyber coupling pathway while keeping the shield. It tests whether the gains of the full model come from selective coupling or simply from safer communication.
- **No targeting + Shield**: an ablation that disables community-level targeting while keeping the shield and forcing broadcast-only communication. It tests whether targeted correction is actually useful relative to broad communication in a given scenario.

The policy set supports four central comparisons. First, Pipeline versus Pipeline+Shield isolates the effect of runtime governance on a sequential baseline. Second, V-CHIMERA versus V-CHIMERA+Shield shows what shielding changes when coupling is present. Third, V-CHIMERA+Shield versus V-CHIMERA+IGC+Shield is the direct test of whether immune gating improves on naive coupling. Fourth, V-CHIMERA+IGC+Shield versus the No coupling+Shield and No targeting+Shield ablations clarifies whether the benefit comes from coupling itself, from targeting, or from the ability to fall back to broader communication.

The policies are defined once and shared across scenarios. We do not retune controller thresholds per scenario. Differences across scenarios arise from the scenario YAML files—for example, network size, bot fraction, moderation rates, initial narrative shocks, and coupling weights—rather than from per-scenario policy tuning. This choice places the burden of generalization on the controller logic instead of on manual scenario-specific adjustment and therefore yields a more credible test of whether the framework behaves robustly across incident types.

### 5.3. Metrics and Statistical Reporting

The primary evaluation uses time-normalized area under the curve (AUC) summaries over the 60-step episode horizon. AUC is useful here because policies can differ not only in their final outcomes but also in how long harmful conditions persist. A policy that allows a short, sharp spike in misbelief may be preferable to one that produces moderate but persistent misbelief over the entire episode, and integrated summaries capture that difference better than endpoint metrics alone.

Our primary operational metric is cyber-harm AUC. On the social side, we report AUC for misbelief, trust, uncertainty, and polarization. Lower is better for cyber harm, misbelief, uncertainty, and polarization, whereas higher is better for trust. Together, these metrics capture the central trade-off in the paper: a controller should reduce operational harm without destabilizing the social environment through unmanaged rumor, uncertainty, or trust collapse.

We also report three protocol-governance metrics: attempted violations, executed violations, and shield edits. Attempted violations count unsafe communication proposals before runtime enforcement. Executed violations count unsafe communications that actually reach the environment after the shield is applied. Shield edits count interventions in which the shield downgrades, rewrites, or blocks the proposed message to satisfy the protocol. This distinction matters operationally. A zero executed-violation result can arise either because the controller already behaved safely or because the shield actively corrected unsafe proposals. Reporting all three quantities makes the safety guarantee auditable rather than purely declarative.

Because AUC alone can obscure endpoint behavior, we also report secondary metrics derived from the same run logs. These include final cyber harm, final misbelief, final trust, exfiltration-risk AUC, and compromised-fraction AUC. These secondary outcomes are not separate experiments; they are alternative summaries of the same episode traces and test whether the IGC-versus-naive-coupling comparison is robust to the choice of metric.

Each scenario–policy pair is evaluated over eight random seeds. With three scenarios and seven policies, the primary evaluation therefore comprises 168 episode runs. All main tables report mean ± one standard deviation over seeds. We use exact paired sign-flip permutation tests when comparing V-CHIMERA+IGC+Shield against V-CHIMERA+Shield, because that is the comparison most directly tied to the central mechanistic claim of the paper: whether immune gating improves on naive coupling under otherwise identical shielding and environment conditions. Because these tests use eight paired seeds and are reported across multiple scenarios and outcomes, the *p*-values are unadjusted and provide exploratory evidence for the mechanistic contrast rather than confirmatory family-wise inference. The interpretation therefore emphasizes effect sizes, directions of change, and consistency across secondary metrics alongside the exact *p*-values.

### 5.4. Shared Defaults, Calibration, and Reproducibility

The controller thresholds and shield settings used in the experiments are shared defaults, reported in Table 3. These include the verification threshold, uncertainty gate, danger threshold, immune-memory decay, antigen threshold, and the core shield cooldown and severity gates. We keep those defaults fixed across all three journal-main scenarios. Only the environment and coupling YAML files vary by scenario. This design choice makes the evaluation conservative: the controller is not hand-tuned separately for ransomware, outage, and exfiltration settings.

The social model is intentionally stylized rather than tied to one platform API or one organization’s telemetry stream. We therefore adopt a calibration-first strategy. In the artifact, the scenario YAML files specify the cyber environment, social graph, bot rate, moderation parameters, and coupling scales, while the controller defaults remain shared. This separation allows the evaluation to isolate controller behavior from scenario design. It also better reflects how the framework would be used in practice: an organization would calibrate the environment-facing parameters to its incident context, but it should not need to redesign the controller logic for every new event type.

To support reproducibility and accessibility, the repository exposes the exact configuration used here through the journal_main experiment and provides scripts that regenerate the tables and figures from a completed run directory. The artifact supports incremental evaluation. Pipeline+Shield provides a conservative reference because it preserves hard communication safety while remaining easy to interpret. The principal coupled evaluation point is V-CHIMERA+IGC+Shield, followed by the No coupling+Shield and No targeting+Shield ablations, which indicate whether a deployment setting behaves more like a localized-misinformation regime or a broadcast-dominant regime.

In short, the experimental setup is designed for controlled comparison rather than scenario-specific optimization. The same policy logic is subjected to three qualitatively different cyber–social environments, evaluated with both outcome and governance metrics, and reported in a form that can be reproduced directly from the released configuration and run pipeline.

## 6. Results

**Figure 2:**
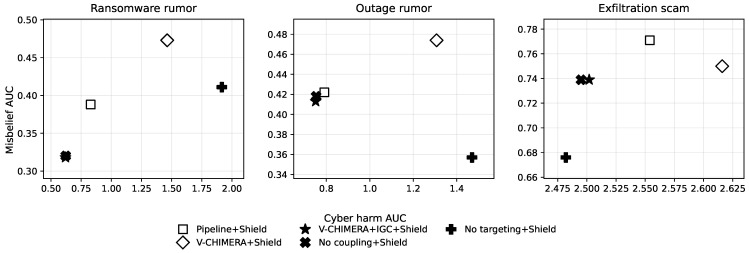
Cyber–social trade-offs for protocol-safe policies by scenario. Each marker shows the mean cyber-harm AUC (*x*-axis) and mean misbelief AUC (*y*-axis) over eight seeds; lower-left is better on both axes. Only shielded policies are shown, and Table 4 reports the corresponding mean ± SD values. In ransomware rumor, V-CHIMERA + IGC + Shield and No coupling + Shield lie on the lower-left frontier. In outage rumor, V-CHIMERA + IGC + Shield achieves the lowest cyber harm, whereas No targeting + Shield yields the lowest misbelief. In exfiltration scam, No targeting + Shield outperforms V-CHIMERA + IGC + Shield on both axes.

**Figure 3:**
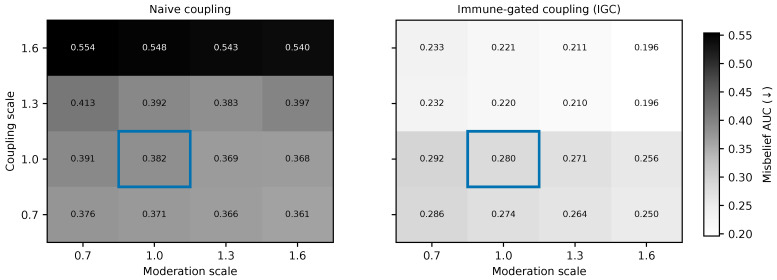
Sensitivity of misbelief AUC in the ransomware rumor scenario as a function of cyber–social coupling and platform moderation. (**left**) naive coupling (V-CHIMERA + Shield). (**right**) immune-gated coupling (V-CHIMERA + IGC + Shield). The blue outline marks the default operating point used in the main experiments: coupling scale = 1.0 and moderation scale = 1.0.

**Figure 4:**
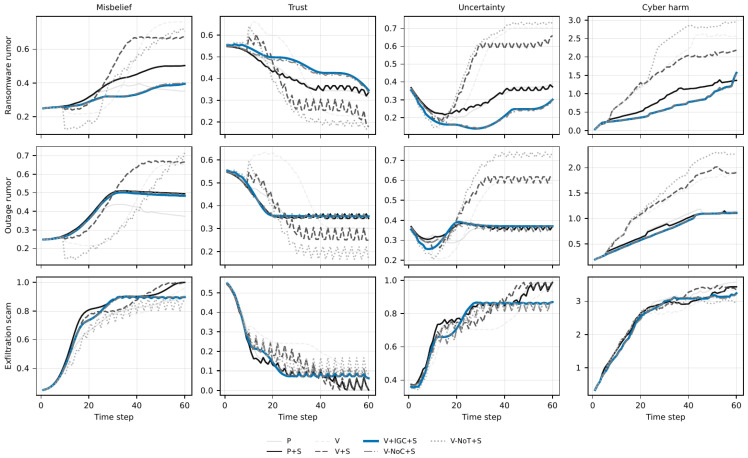
Macro-dynamics over time (mean across seeds). Rows correspond to scenarios; columns show misbelief, trust, uncertainty, and cyber harm. The highlighted accent line corresponds to V-CHIMERA + IGC + Shield, while the remaining lines show baselines and ablations. The immune-gated controller improves stability in ransomware rumor and outage rumor while preserving the zero-violation guarantee for executed communications.

**Figure 5:**
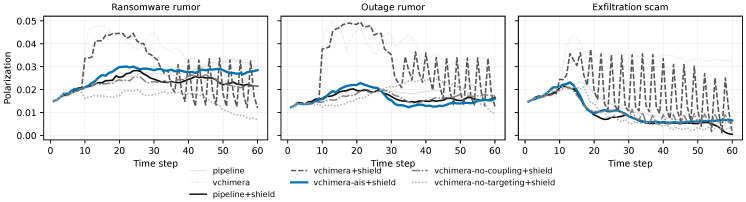
Polarization dynamics over time (mean across seeds) for the three primary scenarios. Legend labels correspond to artifact policy identifiers: vchimera-ais+shield denotes V-CHIMERA + IGC + Shield, vchimera-no-coupling+shield denotes No coupling + Shield, and vchimera-no-targeting+shield denotes No targeting + Shield.

The results are organized in six subsections. Section 6.1 summarizes the main cyber and social outcomes together with the protocol-safety signals (Table 4 and Table 5). Section 6.2 interprets the shielded cyber–social trade-offs (Figure 2). Section 6.3 isolates the contribution of immune gating using paired sign-flip tests (Table 6). Section 6.4 checks whether the IGC-versus-naive-coupling pattern persists under secondary metrics beyond AUC (Table 7). Section 6.5 evaluates robustness under stronger coupling and weaker moderation (Figure 3 and Table 8). Section 6.6 examines the temporal trajectories that generate the aggregate AUC summaries (Figure 4 and Figure 5).

### 6.1. Main Outcomes and Protocol Safety

Table 4 reports the primary cyber and social outcomes, while Table 5 isolates the communication-governance signals. Taken together, the two tables answer RQ3 directly and provide the first evidence relevant to RQ1.

The clearest result is that the runtime shield delivers the intended hard protocol-safety guarantee. Across all three scenarios, every shielded policy executes zero protocol-unsafe communications with respect to the encoded predicates, whereas the unshielded baselines execute frequent violations. This difference is not merely formal. The shield is actively intervening rather than passively preserving safety through silence. For example, in the exfiltration-scam scenario, Pipeline+Shield records 146.125 attempted violations and 47.625 shield edits, while V-CHIMERA+IGC+Shield still requires 35.000 edits despite executing zero protocol-unsafe actions. Likewise, in ransomware rumor, V-CHIMERA+IGC+Shield reduces the number of attempted violations relative to V-CHIMERA+Shield (11.750 vs. 25.750) while preserving the same zero-executed-violation guarantee. The protocol layer therefore matters in two ways: it blocks unsafe messages, and it makes controller behavior auditable through attempted violation and edit logs.

On effectiveness, the results separate *naive* coupling from *regulated* coupling. The naive coupled controller is brittle. In ransomware rumor, V-CHIMERA + Shield performs substantially worse than Pipeline + Shield on cyber harm (1.463 vs. 0.829) and misbelief (0.473 vs. 0.388), and it also yields lower trust (0.369 vs. 0.415). A similar pattern appears in outage rumor, where naive coupling increases cyber-harm AUC from 0.791 to 1.307 and misbelief AUC from 0.422 to 0.474 relative to the shielded pipeline baseline. These results show that simply wiring sentiment into operational response is not enough; when social signals are noisy, delayed, or only weakly verified, naive coupling can amplify rather than reduce incident harm.

Immune-gated coupling improves primarily by preventing that failure mode. In ransomware rumor, V-CHIMERA + IGC + Shield lowers cyber-harm AUC from 1.463 to 0.620 relative to V-CHIMERA + Shield, lowers misbelief from 0.473 to 0.318, and raises trust from 0.369 to 0.470. In outage rumor, the same qualitative pattern holds with smaller but still meaningful gains: cyber harm falls from 1.307 to 0.751, misbelief from 0.474 to 0.413, and trust rises from 0.361 to 0.392. These results support RQ1 in a conditional rather than absolute sense: coupling helps when it is danger-gated and uncertainty-aware, and it can fail when it is not.

It is also important not to overstate what immune gating is doing in the current evaluation. In both ransomware rumor and outage rumor, No coupling + Shield is nearly tied with V-CHIMERA + IGC + Shield on the primary metrics. In ransomware rumor, the two policies differ by only 0.002 in cyber-harm AUC and 0.002 in misbelief AUC; in outage rumor, the difference in cyber-harm AUC is likewise only 0.002. This suggests that, under the present defaults, the main advantage of IGC is not that it always extracts large additional value from social-to-cyber feedback, but that it selectively suppresses harmful coupling and behaves like an adaptive fallback when sentiment information is too noisy to trust.

The exfiltration-scam scenario is more difficult and more revealing. Here, V-CHIMERA+IGC+Shield still improves modestly over the naive coupled controller (2.502 vs. 2.616 cyber-harm AUC; 0.739 vs. 0.750 misbelief AUC), but it is not the best shielded policy. The strongest shielded result is instead the broadcast-only ablation, No targeting+Shield, which attains the lowest cyber harm (2.482), the lowest misbelief (0.676), and the highest trust (0.194) among the shielded policies. This pattern indicates that targeting is not a universal best practice.

Finally, the standard deviations in Table 4 and Table 5 are substantively informative rather than incidental. Outcome variability is largest in the unshielded and sequential baselines, especially in ransomware rumor and outage rumor, because rumor timing, evidence arrival, and defender response interact stochastically. Shielded policies collapse executed-violation variance to zero, but they do not eliminate outcome variance. Exfiltration scam remains comparatively variable even under shielding, which suggests that scam diffusion and trust collapse are strongly path dependent and harder to stabilize than the rumor scenarios.

### 6.2. When Targeting Helps and When It Fails

To keep the trade-off readable, Figure 2 restricts attention to the protocol-safe policies. This removes the confound of executed protocol violations and makes the cyber–social frontier easier to interpret. The figure also makes a point that is less obvious from the tables alone: the effect of targeting is scenario dependent rather than uniform.

In ransomware rumor, V-CHIMERA + IGC + Shield and No coupling + Shield lie on the lower-left frontier and are nearly tied. This indicates that the immune gate is doing its intended job: it avoids the large degradation caused by naive coupling and behaves conservatively when the social signal is not yet reliable enough to justify stronger intervention. In this regime, the main value of IGC is selective activation rather than aggressive exploitation of social-to-cyber feedback.

In outage rumor, the picture is more mixed. V-CHIMERA + IGC + Shield attains the lowest cyber-harm AUC among shielded policies, whereas No targeting + Shield achieves the lowest misbelief AUC. That pattern is operationally plausible. Broadcast-only communication can be effective when many users are affected by the same ambiguous event and benefit from broad clarification, but immune-gated coupling still helps on the cyber dimension by avoiding premature or noisy escalation.

In exfiltration scam, the interpretation becomes sharper: the broadcast-only ablation dominates the targeted immune-gated variant on both axes. No targeting + Shield improves on V-CHIMERA + IGC + Shield in cyber harm (2.482 vs. 2.502) and, more clearly, in misbelief (0.676 vs. 0.739). In other words, when the threat is broadly relevant and follow-on victimization depends heavily on rapid mass reporting, segmentation can slow the response too much to be beneficial.

This pattern has a practical interpretation. Targeting is most useful when misinformation risk is concentrated, community boundaries remain meaningful, and messaging capacity is limited enough that broad communication would dilute attention. Targeting is less useful when the event is broadly salient, when risk is widely shared, or when misinformation crosses communities faster than a segmented strategy can react. The exfiltration-scam scenario is the clearest example: wide-audience communication increases reporting and reduces scam exposure more effectively than narrow correction. The empirical lesson is therefore not that targeting is abandoned, but that targeting functions as a conditional design choice rather than a universal default.

### 6.3. Paired Comparison of IGC Against Naive Coupling

To isolate the value of immune-style regulation itself, Table 6 compares V-CHIMERA + IGC + Shield directly against V-CHIMERA + Shield using exact paired sign-flip permutation tests across seeds. Because the tests are unadjusted and use a small paired sample, they support a mechanism-level interpretation rather than confirmatory family-wise inference. The paired design is useful because it evaluates whether immune gating produces consistent within-seed changes rather than isolated favorable seeds.

The paired tests provide exploratory evidence of reductions in cyber harm for the two rumor scenarios. In ransomware rumor, the mean paired difference in cyber-harm AUC is −0.843 with *p* = 0.031; in outage rumor, it is −0.556 with *p* = 0.047. These are the strongest inferential signals in the paper, but the effect sizes and secondary metrics remain central because the tests are not multiplicity-adjusted confirmatory findings. Under that interpretation, they support the central claim that immune gating protects against the brittleness of naive coupling.

The corresponding changes in misbelief and trust are directionally favorable in the rumor scenarios but are not distinguishable at this sample size. In ransomware rumor, misbelief decreases by 0.155 and trust increases by 0.101 on average, but the paired *p*-values remain above conventional thresholds. In outage rumor, the average improvements are smaller still. This is not inconsistent with the design objective of IGC. The controller is primarily a regulator intended to prevent overreaction and stabilize coupling under uncertainty; it is not expected to dominate every social metric in every run.

The exfiltration-scam scenario again behaves differently. The paired differences between immune-gated and naive coupled control are small and not statistically distinguishable across all three primary outcomes. This is consistent with the broader picture from Section 6.2: the main issue in that scenario is not merely that naive coupling is unstable, but that targeted control itself is less appropriate than broad communication.

### 6.4. Secondary Metrics Beyond AUC

Table 7 reports final-state outcomes and decomposed cyber metrics for naive coupled control and immune-gated coupling. These additional metrics test whether the observed advantage of immune-gated coupling over naive coupled control is robust beyond integrated AUC summaries. The same qualitative pattern persists across final-state and decomposed cyber metrics. In both ransomware rumor and outage rumor, immune-gated coupling lowers final harm, lowers final misbelief, improves final trust, reduces exfiltration-risk AUC, and lowers compromised exposure relative to the naive coupled controller. In ransomware rumor, for example, final harm decreases from 2.184 to 1.569 and exfiltration-risk AUC drops from 0.410 to 0.142. In outage rumor, final harm decreases from 1.904 to 1.113 and compromised AUC falls from 0.143 to 0.086. These improvements show that the benefit of IGC is not limited to one summary statistic; it appears in both aggregate trajectories and endpoint outcomes.

The exfiltration-scam scenario remains more resistant to improvement, but even there the secondary metrics are consistent with the main story. Relative to naive coupled control, V-CHIMERA + IGC + Shield modestly lowers final harm (3.345 to 3.238), lowers final misbelief (0.999 to 0.898), raises final trust (0.002 to 0.062), and reduces both exfiltration risk and compromised exposure. These gains show that immune gating still improves on naive coupling, even though the broadcast-only ablation remains the strongest overall shielded policy in this scenario.

Taken together, Table 7 strengthens the interpretation of the main tables. The IGC-versus-naive-coupling result does not depend on a particular way of aggregating trajectories: immune-gated coupling improves stability across multiple operationally meaningful views of the same runs.

### 6.5. Robustness and Sensitivity

We next vary cyber–social coupling strength and platform moderation in the ransomware-rumor scenario. This experiment asks whether the gains from immune gating depend on a narrow operating point or whether they persist as the environment becomes more tightly coupled and more socially permissive.

Figure 3 shows a clear asymmetry between naive and immune-gated coupling. Under naive coupling, misbelief AUC rises as coupling strengthens and moderation weakens, reaching its worst values in the high-coupling/low-moderation corner. Under immune-gated coupling, the corresponding surface is flatter and consistently lower. The difference is especially informative because the two policies share the same underlying environmental sweep; what changes is the control logic that decides when social feedback is trusted and how escalation is triggered.

Table 8 makes the effect explicit at two extreme settings. Under low coupling and high moderation, naive coupling yields a misbelief AUC of 0.361 whereas immune-gated coupling yields 0.250, a gap of 0.111. Under high coupling and low moderation, the gap widens substantially: naive coupling rises to 0.554 while immune-gated coupling remains at 0.233, for a difference of 0.321. The widening gap is the key result. It shows that IGC is not merely a better-tuned coupled controller at one setting; it is a more stable way to use coupling when the cyber and social layers become more tightly interdependent and platform-level filtering weakens.

This robustness result also clarifies the role of immune gating conceptually. The controller is not valuable because it always extracts more information from the social layer. It is valuable because it prevents harmful amplification in exactly the settings where naive coupling would most likely overreact to noisy or adversarially shaped sentiment signals.

### 6.6. Macro-Dynamics over Time

The aggregate AUC summaries are useful, but they do not show *when* the policies diverge over the course of an episode. Figure 4 and Figure 5 therefore report the mean temporal trajectories for the three primary scenarios.

The time-series plots are consistent with the main aggregate results. In ransomware rumor, immune-gated coupling avoids the large overshoot seen under naive coupling: misbelief grows more slowly, trust remains higher, uncertainty is damped earlier, and cyber harm accumulates less steeply. Outage rumor shows a similar but weaker pattern. The main benefit again appears to be stabilization: IGC reduces the amplitude of the social and cyber swings that otherwise arise when the controller reacts too directly to noisy social signals.

Exfiltration scam behaves differently. Across most policies, misbelief rises rapidly, trust falls sharply, and cyber harm accumulates more persistently than in the rumor scenarios. This is consistent with the higher variability reported earlier and with the finding that broadcast-only communication outperforms targeted messaging in this setting. The temporal plots therefore help explain why the aggregate AUC improvements are smaller here: the scenario is driven by fast, broad diffusion and a narrow window for effective intervention.

Figure 5 provides a complementary view of social stability. In ransomware rumor and outage rumor, the immune-gated and no-coupling shielded policies generally track lower or comparable polarization than naive coupling, especially during the middle part of the episode when public interpretation of the incident is still unstable. In exfiltration scam, polarization also becomes more volatile early on, then decays later as the crisis saturates the population. These patterns reinforce the broader conclusion that IGC mainly helps by preventing destabilizing feedback loops rather than by maximizing any single metric in isolation.

Taken together, the temporal trajectories explain the main story of the section. The runtime shield guarantees protocol safety for executed communications, while immune gating reduces the risk that noisy social feedback will destabilize cyber response. When the scenario rewards selective coupling, as in the rumor cases, this produces clear gains. When the scenario favors fast population-wide communication, as in exfiltration scam, the main lesson is instead that broadcast can be the more appropriate policy.

## 7. Discussion, Deployment Guidance, and Limitations

The results in Table 4, Table 5, Table 6, Table 7 and Table 8 and Figure 2, Figure 3, Figure 4 and Figure 5 support a restrained interpretation of coupled cyber–social control. The main value of V-CHIMERA is not universal dominance across all scenarios. Rather, the value lies in making coupling safer to use, easier to audit, and less brittle under uncertainty. The runtime shield addresses the governance problem by eliminating executed violations with respect to the encoded communication protocol, while the immune-gated controller addresses the control problem by preventing premature reliance on noisy social signals. The ablations then clarify a third issue that matters in practice: targeting is useful in some regimes, but it is not a universal default. These empirical patterns inform the deployment guidance and limitations discussed below.

### 7.1. Practical Interpretation of Targeting and Broadcast

The clearest operational lesson from Figure 2 and Table 4 is that targeting is a *regime-dependent* policy choice. Targeted communication is most attractive when misinformation risk is concentrated within a small number of communities, when those communities differ meaningfully in susceptibility or trust, and when messaging capacity is scarce enough that broad communication would dilute attention or create unnecessary fatigue. In that regime, the immune-memory component is useful because it suppresses one-step noise spikes and waits for persistent local antigen load before escalating toward narrower corrective action.

By contrast, targeting can fail for structural rather than merely algorithmic reasons. The exfiltration-scam scenario illustrates a case in which the threat is broadly relevant, the benefit of rapid mass reporting is high, and misinformation can diffuse across community boundaries faster than a segmented policy can react. In such settings, broadcast messaging does not simply reach more people; it can also improve the cyber side of the problem by increasing reporting, accelerating detection, and reducing the time window in which follow-on fraud can spread. A narrowly targeted policy may therefore be too slow even if its community ranking is locally correct.

A practical implication follows from this pattern. In the earliest phase of a high-salience incident, broad transparency, explicit uncertainty labeling, and requests for reports are appropriate defaults. As evidence stabilizes and immune-memory estimates identify persistent local pockets of misbelief, messaging can narrow toward targeted corrections or community-specific advisories. The controller then reverts to broader communication whenever aggregate misbelief rises above the broadcast-fallback threshold, trust falls sharply, or the number of affected communities grows. In other words, targeting is best treated as a second-stage refinement of communication strategy rather than as a universal first move. This regime-dependent conclusion is also consistent with crisis-correction research showing that correction design, detail level, and perceived corrective quality shape whether organizational counter-messaging is accepted or ignored [16,17,18].

### 7.2. External Validity and Empirical Calibration

External validity depends on whether the abstract variables in the simulator can be mapped to audited organizational signals. On the cyber side, incident severity can be estimated from service degradation, asset criticality, compromise scope, exfiltration indicators, restoration backlog, and response cost. Detection confidence can be derived from analyst-validated evidence, agreement across sensors, or triage-confidence scores. On the social side, misbelief, trust, uncertainty, compliance, and reporting are not directly observable and must therefore be approximated using proxies such as help-desk traffic, phishing-report volume, click-through to official advisories, customer-support topics, survey instruments, or sentiment-monitoring pipelines that are periodically checked against human review. Likewise, community structure may be defined by business units, customer segments, geographic regions, platform clusters, or graph communities, provided that the resulting segmentation reflects how information and guidance actually flow through the organization.

Calibration is therefore staged. Retrospective incidents or tabletop exercises can be used to fit the coupling and shock parameters so that the simulator reproduces coarse historical trajectories, after which the controller can be stress-tested across plausible parameter ranges before any live operational use. The parameters most likely to require empirical estimation are the cyber-to-social and social-to-cyber coupling scales, trust-loss and uncertainty-shock coefficients, correction efficacy, official-message efficacy, moderation strength, bot prevalence, and segmentation quality. The shared defaults in Table 3 are best interpreted as research defaults and conservative initial settings rather than as deployment-optimal values.

Equally important, the runtime shield requires organization-specific governance predicates. The current protocol covers cooldowns, evidence requirements, uncertainty labeling, and severity gates, but real deployments may need additional constraints tied to legal review, privacy obligations, sector regulation, approval chains, and brand-risk policies. The guarantee delivered by the shield is only as strong as the predicate set it enforces. Predicate design is therefore a joint security, communications, legal, and risk responsibility rather than a purely technical configuration.

### 7.3. Artifact Configuration and Operational Defaults

The artifact supports both methodological replication and operational prototyping. For methodological replication, the repository’s journal_main configuration reproduces the same three scenarios and seven policies used in this paper. The Pipeline+Shield versus V-CHIMERA+IGC+Shield contrast isolates the value of joint cyber–social control while preserving protocol safety. The No coupling+Shield and No targeting+Shield ablations then indicate whether the deployment setting behaves more like a localized-misinformation regime or a broad-salience regime.

For operational prototyping, three defaults are especially useful anchors: a verified-detection threshold around 0.60, an uncertainty gate around 0.55, and a danger threshold around 0.65. Together with the default cooldown, these settings create a conservative posture in which coupled control activates only when cyber evidence is credible and social signals are informative enough to trust. This reduces the chance that noisy or manipulated sentiment will drive cyber operations too early, while still allowing broad transparency and reporting solicitation during ambiguous phases of an incident.

These defaults can then be calibrated according to organizational risk tolerance and incident type. High-liability environments may require stricter evidence thresholds, longer cooldowns, and more aggressive downgrade rules in the shield. Scam-heavy consumer contexts may instead benefit from preserving strict debunk requirements while making reporting-solicitation behavior more permissive. The ablations serve as diagnostic tools rather than merely research baselines: if No coupling + Shield matches or outperforms V-CHIMERA + IGC + Shield, then the organization probably lacks enough calibrated signal to justify operational coupling; if No targeting + Shield consistently outperforms targeted control, then the segmentation or diffusion assumptions are likely too weak for narrow messaging to add value.

### 7.4. Limitations

This study has several limitations. First, the cyber backend is an abstract incident simulator rather than a live enterprise environment, and the social backend is a stylized agent-based model rather than a platform-specific behavioral system. The framework does not represent natural-language semantics, media narratives, recommender algorithms, or cross-platform migration in full detail. It also simplifies the social-to-cyber interface by passing aggregate compliance and reporting propensities through linear modifiers. These abstractions are deliberate because they make controlled comparison, reproducibility, and mechanism-level interpretation possible, but they limit how directly the numerical results should be extrapolated to a specific organization.

Second, the evaluated controllers are transparent rule-based policies rather than learned, model-predictive, or game-theoretic policies. This is a strength for interpretability and auditability, but it positions the results as evidence about control structure rather than proof of optimality. A stronger learned controller paired with the same shield might outperform the present policies, and a strategic adversary might adapt its messaging tactics in response to the organization’s communication behavior in ways that are not captured here.

Third, the safety guarantee is scoped to the encoded protocol predicates. The shield guarantees zero executed violations only with respect to those predicates. It does not guarantee the absence of all communication harm, because harmful but rule-compliant messages can still exist if the rule set is incomplete, mis-specified, or insufficiently tailored to the organization’s legal and reputational constraints. Likewise, community targeting depends on segmentation quality. If communities are poorly estimated, under-observed, or socially inappropriate, targeted intervention can miss affected groups, create inequitable coverage, or appear arbitrary to both operators and audiences.

Finally, the biomimetic analogy and application scope are narrow. The immune metaphor is used as a functional design vocabulary for escalation, damping, persistence, and checkpointing; V-CHIMERA is not a biological simulation, and the value of the analogy lies in the controller structure it helps organize rather than in biological fidelity. In addition, the present study focuses on acute organizational crises, not state-level information operations, long-horizon influence campaigns, or platform-wide governance. Future work can combine the same coupling abstraction and verified shielding layer with richer social semantics, online parameter estimation, human-in-the-loop approval workflows, and learned multi-agent policies that preserve runtime safety.

## 8. Conclusions

We presented V-CHIMERA, an immune-inspired verified framework for organizational cyber crisis response under misinformation. Rather than treating incident response and public communication as separate workflows, V-CHIMERA models them as coupled control loops, enforces hard communication constraints through a runtime shield, and uses immune-gated coupling to regulate when social feedback should influence cyber operations and how strongly counter-messaging should escalate.

Three conclusions emerge from the evaluation. First, protocol safety can be enforced at execution time: every shielded policy executed zero protocol-unsafe communications with respect to the encoded predicates across the evaluated scenarios. Second, cyber–social coupling is not beneficial by default. Naive coupling proved brittle, whereas immune-gated coupling substantially reduced cyber harm relative to naive coupled control in ransomware rumor and outage rumor while also improving or preserving social outcomes. This stabilizing role is reinforced by the sensitivity analysis, which showed that immune-gated coupling attenuates degradation under stronger coupling and weaker moderation. Third, targeted messaging is a conditional strategy rather than a universal best practice. In the exfiltration-scam scenario, broadcast-only communication outperformed targeted messaging, indicating that broad communication can be preferable when risk is widely shared and misinformation diffuses rapidly across communities.

Taken together, these results suggest that the value of biomimetic regulation lies not in guaranteeing universal superiority, but in making coupled cyber–social control more disciplined, selective, and auditable. By combining danger-triggered escalation, tolerance-style gating, immune memory, and hard runtime checks, V-CHIMERA provides a practical template for misinformation-aware organizational incident response. Future work can test these ideas with empirically calibrated organizational data, richer language and platform dynamics, and learned controllers that retain the same runtime safety guarantees.

## Figures and Tables

**Table 1:** Selected related work, emphasizing recent studies while also including foundational references directly relevant to crisis communication, multi-agent coordination, and the biomimetic controller.

Study	Methodology/Focus	Representative Contribution	Limitation Relative to this Work
Carley [1]; Mulahuwaish et al. [2]	Conceptual and survey work on social cybersecurity	Establish that cyber outcomes depend on human behavior, social influence, and online information flows	Do not specify a closed-loop organizational incident-response controller with explicit communication governance
Austin et al. [3]; Cheng et al. [4]; Shahbazi and Bunker [5]	Social-mediated crisis communication and crisis-information trust	Show that publics seek crisis information across official and social channels and that trust shapes crisis response	Study communication and trust outcomes, not closed-loop cyber-operational incident response
Kim and Lim [16]; Lu and Jin [17,18]	Organizational corrective communication and prebunking in crises	Show that correction strategy, timing, detail, and perceived corrective quality shape crisis-misinformation response	No coupled cyber backend, operational incident metrics, or runtime communication governance
Lazer et al. [10]; Vosoughi et al. [11]; Pennycook and Rand [12]; Lewandowsky and van der Linden [13]; Ng et al. [14]; Kozyreva et al. [15]	Misinformation diffusion, susceptibility, and intervention studies	Explain false-news diffusion, cognitive susceptibility, prebunking, moderation, and individual-level intervention design	Evaluate information outcomes rather than operational cyber outcomes or cyber–social feedback loops
Baillie et al. [6]; Standen et al. [7]; Emerson et al. [8]; Kunz et al. [9]	Autonomous cyber-defense simulation environments	Provide enterprise-scale benchmarks for defender policies under uncertainty	Omit public communication, trust, misinformation dynamics, and communication governance
Wooldridge [26]; Jennings [27]; Rahwan et al. [28]; Tambe [29]	Multi-agent coordination, machine behavior, and security decision-making	Provide foundations for coordination among autonomous agents, algorithmic social dynamics, and adversarial resource allocation	Do not model governed crisis communication or closed-loop cyber–social incident response
Alshiekh et al. [19]; Koenighofer et al. [20]	Runtime shielding for safe reinforcement learning	Show that hard runtime constraints can guarantee safe executed actions	Not designed for misinformation-aware organizational crisis communication with sentiment-coupled cyber response
Greensmith et al. [24]; de Castro and Timmis [25]	Artificial immune systems and danger-theoretic adaptive regulation	Introduce danger signaling, immune memory, tolerance thresholds, and graded response for adaptive control under uncertainty	Provide biomimetic control ideas, but not a shielded cyber–social incident-response architecture for organizational crises

**Table 2:** Functional mapping from immune-system concepts to the implemented V-CHIMERA + IGC controller. The mapping is used as a design analogy rather than a literal biological equivalence.

Immune Mechanism	Realization in V-CHIMERA + IGC	Design Role
Danger signaling	Weighted combination of incident severity and detection-confidence gap	Activates coupled control only when cyber evidence warrants escalation
Antigen recognition	Community risk score from misbelief, low trust, and uncertainty	Identifies subpopulations most at risk of persistent misinformation
Immune memory	Exponential moving average over community antigen load	Stabilizes targeting and suppresses one-step noise spikes
Tolerance threshold	Danger/uncertainty gate for coupling and antigen threshold for targeting	Prevents premature reliance on noisy sentiment signals
Clonal expansion	Messaging intensity increases with persistent antigen load	Scales response strength to sustained rather than transient risk
Broadcast fallback	Elevated macro misbelief or low trust switches targeting to all communities	Avoids over-segmentation when the crisis becomes broadly salient
Immune checkpoints	Runtime communication shield with hard protocol predicates	Prevents unsafe public messages from executing and logs edits for auditability

**Table 3:** Shared controller and shield defaults used across all journal-main scenarios. Only the environment and coupling YAML files differ by scenario; the policy thresholds below were not retuned per scenario.

Symbol/Setting	Default	Role	Rationale
$\theta_{\det}$	0.60	Detection confidence treated as sufficiently verified	Aligned with the protocol evidence threshold so policy escalation and shield verification are consistent
$\tau_{u}$	0.55	Maximum uncertainty for danger-gated coupling	Matches the uncertainty-label threshold used by the shield; above this level the controller remains conservative
$\tau_{d}$	0.65	Danger threshold for enabling coupled cyber control	Slightly stricter than $\theta_{\det}$ to avoid overreacting to weak evidence
δ	0.85	Immune-memory decay in Equation (8)	Retains context from recent steps while still incorporating new evidence (15% new mass per update)
$\tau_{a}$	0.35	Minimum antigen load for community targeting	Prevents low-signal communities from being targeted
$\left(w_{M},w_{T},w_{U}\right)$	(1.00,0.80,0.30)	Antigen weights in Equation (7)	Prioritizes persistent misbelief and trust erosion while letting uncertainty contribute as a secondary risk cue
$\left(w_{\mathrm{sev}},w_{\mathrm{gap}}\right)$	(0.70,0.30)	Danger weights in Equation (5)	Gives more weight to realized severity than to lack of verification alone
$I_{0},\kappa$	0.35,0.45	Base intensity and clonal gain in Equation (9)	Starts with moderate communication and scales upward only when persistent community risk accumulates
$\theta_{M},\theta_{T}$	0.22,0.45	Aggregate misbelief and trust thresholds for debunk/broadcast fallback logic	Triggers evidence-based debunks only when misbelief is meaningfully elevated and forces all-audience communication when the crisis becomes broadly salient or trust is already fragile
Cooldown/severity gate	3 steps/0.75	Hard protocol constraints	Conservative communication defaults that suppress rapid repeated messaging and disallow debunking during high-severity, weak-evidence phases

**Table 4:** Main results (mean ± SD over seeds, *n* = 8). Lower is better for cyber harm and misbelief; higher is better for trust. Arrows indicate the preferred direction (down: lower is better; up: higher is better). “Exec. viol.” counts executed protocol violations (0 for all shielded policies). Boldface indicates the best shielded result per scenario for each primary metric.

Scenario	Policy	Cyber Harm AUC ↓	Misbelief AUC ↓	Trust AUC ↑	Exec. Viol. ↓	Shield Edits ↓
Exfiltration scam	Pipeline	2.648 ± 0.867	0.688 ± 0.172	0.134 ± 0.124	162.750 ± 56.188	0.000 ± 0.000
	Pipeline + Shield	2.554 ± 0.768	0.771 ± 0.187	0.137 ± 0.124	0.000 ± 0.000	47.625 ± 15.296
	V-CHIMERA	2.454 ± 0.975	0.694 ± 0.254	0.238 ± 0.174	48.625 ± 18.244	0.000 ± 0.000
	V-CHIMERA + Shield	2.616 ± 0.754	0.750 ± 0.188	0.166 ± 0.143	0.000 ± 0.000	35.500 ± 8.036
	V-CHIMERA + IGC + Shield	2.502 ± 1.033	0.739 ± 0.226	0.155 ± 0.173	0.000 ± 0.000	35.000 ± 13.607
	No coupling + Shield	2.495 ± 1.022	0.739 ± 0.225	0.165 ± 0.164	0.000 ± 0.000	32.500 ± 12.773
	No targeting + Shield	**2.482 ± 1.015**	**0.676 ± 0.261**	**0.194 ± 0.198**	0.000 ± 0.000	33.125 ± 12.311
Outage rumor	Pipeline	0.819 ± 1.067	0.365 ± 0.217	0.398 ± 0.207	56.250 ± 78.305	0.000 ± 0.000
	Pipeline + Shield	0.791 ± 1.016	0.422 ± 0.272	0.388 ± 0.221	0.000 ± 0.000	19.750 ± 27.259
	V-CHIMERA	1.251 ± 0.995	0.384 ± 0.188	0.462 ± 0.166	35.500 ± 23.634	0.000 ± 0.000
	V-CHIMERA + Shield	1.307 ± 1.055	0.474 ± 0.257	0.361 ± 0.246	0.000 ± 0.000	25.750 ± 16.637
	V-CHIMERA + IGC + Shield	**0.751 ± 0.921**	0.413 ± 0.278	**0.392 ± 0.240**	0.000 ± 0.000	17.625 ± 18.531
	No coupling + Shield	0.753 ± 0.925	0.418 ± 0.272	0.388 ± 0.226	0.000 ± 0.000	15.875 ± 16.392
	No targeting + Shield	1.470 ± 0.934	**0.357 ± 0.175**	0.316 ± 0.211	0.000 ± 0.000	24.375 ± 12.432
Ransomware rumor	Pipeline	0.829 ± 1.071	0.334 ± 0.169	0.416 ± 0.184	45.375 ± 68.325	0.000 ± 0.000
	Pipeline + Shield	0.829 ± 1.071	0.388 ± 0.235	0.415 ± 0.188	0.000 ± 0.000	15.625 ± 22.665
	V-CHIMERA	1.636 ± 1.097	0.434 ± 0.181	0.423 ± 0.144	37.750 ± 19.984	0.000 ± 0.000
	V-CHIMERA + Shield	1.463 ± 1.074	0.473 ± 0.256	0.369 ± 0.225	0.000 ± 0.000	25.750 ± 16.918
	V-CHIMERA + IGC + Shield	**0.620 ± 0.958**	**0.318 ± 0.205**	**0.470 ± 0.173**	0.000 ± 0.000	11.750 ± 13.285
	No coupling + Shield	0.622 ± 0.963	0.320 ± 0.202	0.460 ± 0.163	0.000 ± 0.000	9.625 ± 11.363
	No targeting + Shield	1.916 ± 1.419	0.411 ± 0.223	0.332 ± 0.204	0.000 ± 0.000	22.875 ± 11.569

**Table 5:** Protocol compliance (mean ± SD over seeds, *n* = 8). Arrows indicate the preferred direction (down: lower is better). The shield prevents unsafe communication actions from executing while logging attempted violations and edits.

Scenario	Policy	Attempted Viol. ↓	Executed Viol. ↓	Shield Edits ↓
Exfiltration scam	Pipeline	162.750 ± 56.188	162.750 ± 56.188	0.000 ± 0.000
	Pipeline + Shield	146.125 ± 50.572	0.000 ± 0.000	47.625 ± 15.296
	V-CHIMERA	48.625 ± 18.244	48.625 ± 18.244	0.000 ± 0.000
	V-CHIMERA + Shield	35.500 ± 8.036	0.000 ± 0.000	35.500 ± 8.036
	V-CHIMERA + IGC + Shield	37.750 ± 18.437	0.000 ± 0.000	35.000 ± 13.607
	No coupling + Shield	32.500 ± 12.773	0.000 ± 0.000	32.500 ± 12.773
	No targeting + Shield	33.125 ± 12.311	0.000 ± 0.000	33.125 ± 12.311
Outage rumor	Pipeline	56.250 ± 78.305	56.250 ± 78.305	0.000 ± 0.000
	Pipeline + Shield	46.750 ± 64.613	0.000 ± 0.000	19.750 ± 27.259
	V-CHIMERA	35.500 ± 23.634	35.500 ± 23.634	0.000 ± 0.000
	V-CHIMERA + Shield	25.750 ± 16.637	0.000 ± 0.000	25.750 ± 16.637
	V-CHIMERA + IGC + Shield	17.625 ± 18.531	0.000 ± 0.000	17.625 ± 18.531
	No coupling + Shield	15.875 ± 16.392	0.000 ± 0.000	15.875 ± 16.392
	No targeting + Shield	24.375 ± 12.432	0.000 ± 0.000	24.375 ± 12.432
Ransomware rumor	Pipeline	45.375 ± 68.325	45.375 ± 68.325	0.000 ± 0.000
	Pipeline + Shield	39.875 ± 60.546	0.000 ± 0.000	15.625 ± 22.665
	V-CHIMERA	37.750 ± 19.984	37.750 ± 19.984	0.000 ± 0.000
	V-CHIMERA + Shield	25.750 ± 16.918	0.000 ± 0.000	25.750 ± 16.918
	V-CHIMERA + IGC + Shield	11.750 ± 13.285	0.000 ± 0.000	11.750 ± 13.285
	No coupling + Shield	9.625 ± 11.363	0.000 ± 0.000	9.625 ± 11.363
	No targeting + Shield	22.875 ± 11.569	0.000 ± 0.000	22.875 ± 11.569

**Table 6:** Exact paired sign-flip permutation tests comparing V-CHIMERA + IGC + Shield to V-CHIMERA + Shield across seeds (*n* = 8). Δ denotes the mean paired difference (IGC minus naive coupled). The reported *p*-values are exact but unadjusted and exploratory. Lower is better for harm and misbelief; higher is better for trust. Arrows indicate the preferred direction (down: lower is better; up: higher is better).

Scenario	Δ Harm	*p*	Δ Misbelief	*p*	Δ Trust	*p*
ransomware_rumor	−0.843	0.031	−0.155	0.125	+0.101	0.211
outage_rumor	−0.556	0.047	−0.061	0.500	+0.031	0.594
exfiltration_scam	−0.114	0.648	−0.011	1.000	−0.011	0.695

**Table 7:** Secondary metrics beyond the primary AUC endpoints, comparing the naive coupled shielded controller to immune-gated coupling. Values are mean ± SD over seeds (*n* = 8). Arrows indicate the preferred direction (down: lower is better; up: higher is better). These metrics are derived from the same run logs and indicate that the observed IGC advantage over naive coupled control is not an artifact of AUC-only reporting.

Scenario	Policy	Final Harm ↓	Final Misbelief ↓	Final Trust ↑	Exfil Risk AUC ↓	Compromised AUC ↓
Ransomware rumor	V-CHIMERA + Shield	2.184 ± 1.186	0.675 ± 0.448	0.177 ± 0.262	0.410 ± 0.324	0.179 ± 0.076
	V-CHIMERA + IGC + Shield	1.569 ± 2.140	0.395 ± 0.374	0.346 ± 0.256	0.142 ± 0.261	0.095 ± 0.059
Outage rumor	V-CHIMERA + Shield	1.904 ± 1.493	0.666 ± 0.461	0.250 ± 0.342	0.371 ± 0.310	0.143 ± 0.078
	V-CHIMERA + IGC + Shield	1.113 ± 1.378	0.484 ± 0.427	0.354 ± 0.291	0.240 ± 0.331	0.086 ± 0.034
Exfiltration scam	V-CHIMERA + Shield	3.345 ± 0.242	0.999 ± 0.001	0.002 ± 0.000	0.720 ± 0.213	0.250 ± 0.023
	V-CHIMERA + IGC + Shield	3.238 ± 0.931	0.898 ± 0.289	0.062 ± 0.169	0.684 ± 0.291	0.225 ± 0.034

**Table 8:** Sensitivity summary at extreme settings for the ransomware rumor scenario. Values report misbelief AUC for the naive coupled controller and the immune-gated controller.

Setting	Naive Coupling	Immune-Gated Coupling (IGC)	Δ (Naive–IGC)
Low coupling/high moderation	0.361	0.250	0.111
High coupling/low moderation	0.554	0.233	0.321

**Figure A1:**
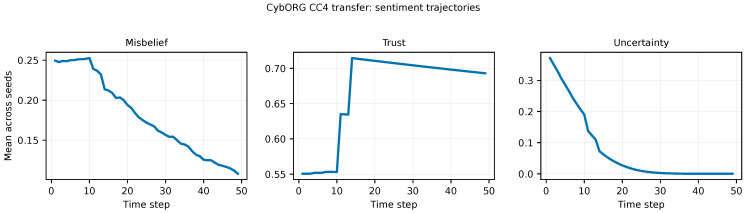
Illustrative CybORG CC4 enterprise transfer run: mean misbelief, trust, and uncertainty trajectories over seeds 0 and 1 from the cyborg_transfer.yaml configuration. The figure is included as an integration check showing that the same cyber–social controller and runtime communication shield can operate when cyber observations are supplied by CybORG rather than by the default incident backend.

**Figure A2:**
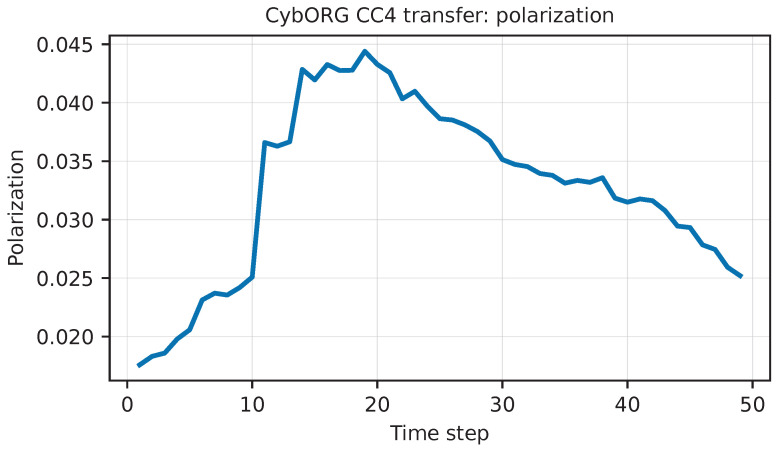
Mean polarization trajectory over seeds 0 and 1 for the illustrative CybORG CC4 enterprise transfer run. This plot is reported as a qualitative stability signal for the transferred loop rather than as a standalone benchmark endpoint.

## Data Availability

The simulation code, configuration files, and paper-generation scripts used in this study are available in the public repository at https://github.com/alqithami/vchimera (accessed on 20 March 2026). The default journal-main experiment configuration reproduces the main study. All data reported here are generated through simulation and can be reproduced from the provided run pipeline.

## Abbreviations

The following abbreviations are used in this manuscript:

ABM	Agent-based model
AUC	Area under the curve
IGC	Immune-gated coupling
RQ	Research question
V-CHIMERA	Verified Coupled Human–Information–Machine Incident Response Architecture

## Appendix A. CybORG Transfer Experiment (Artifact Extension)

The main evaluation in the body of the paper uses the default CyberCrisisGym-J incident backend because it provides a controlled environment for comparing cyber–social policies under identical conditions. A separate question, however, is whether the same framework can be attached to a richer enterprise-defense simulator without redesigning the controller or relaxing the communication-safety guarantees. To examine that question, the artifact includes an adapter for the CybORG ecosystem [6,7,8]. CybORG provides a higher-fidelity enterprise-defense setting with richer cyber observations than the default attack-graph backend.

The appendix has a narrow scope. Its purpose is to verify *portability* of the V-CHIMERA architecture—including the coupling interface, controller logic, and runtime communication shield—to an external cyber-defense backend. It is not a head-to-head CybORG benchmark, and it does not claim superiority over native CybORG policies or baselines. Establishing that kind of benchmark would require a larger scenario suite, repeated seeded evaluation, and calibration specific to the CybORG domain.

### Appendix A.1. Adapter Mapping and Interface Semantics

The adapter acts as an interface layer between CybORG observations and the shared cyber state used by V-CHIMERA. On each step, CybORG-side signals such as service availability, detection alerts, and evidence proxies are mapped into the normalized cyber quantities consumed by the estimator, controller, coupling bus, and protocol monitor. In practice, the mapping preserves the same categories of information used by the default backend: incident severity, verification confidence, service-disruption cues, and indicators relevant to exfiltration or compromise progression. This allows the higher-level controller logic to remain unchanged while the underlying cyber simulator is replaced.

The transfer is intentionally modular. The cyber backend continues to govern enterprise-state evolution, while the social layer interacts with it only through the same abstract channels used elsewhere in the paper: cyber-to-social narrative shocks and social-to-cyber behavior modifiers such as compliance and reporting. As a result, the same communication policy, targeting logic, and runtime shield can be exercised in the CybORG loop once the observation and modifier interfaces are aligned. This is the main architectural point of the adapter: V-CHIMERA is not tied to one specific incident simulator, but to a shared interface between cyber evidence, social dynamics, and governed communication.

### Appendix A.2. Illustrative Transfer Run

For the end-to-end integration check, the artifact includes an illustrative run on the CybORG CC4 enterprise scenario. Figure A1 and Figure A2 are reported as an *integration check* rather than a full empirical benchmark. The run uses the artifact’s configs/experiments/cyborg_transfer.yaml configuration, the cyborg_enterprise_cc4 scenario file, seeds [0,1], a 50-step horizon, and the shielded vchimera+shield policy. The scenario contains 900 social agents and nine communities, and it preserves the same core protocol thresholds used in the main shield defaults: a three-step cooldown, evidence threshold 0.60, uncertainty-label threshold 0.55, and severity gate 0.75. These details make the transfer example reproducible, while the interpretation remains limited to showing that the same closed-loop architecture used in the main experiments remains operational when the cyber observations originate from CybORG rather than from the default attack-graph backend.

The qualitative trajectories are consistent with a functioning transfer. As shown in Figure A1, misbelief declines over time, trust rises and then stabilizes, and uncertainty decays as the episode progresses. These patterns indicate that the social layer remains responsive when driven by an external cyber backend rather than the default simulator. Figure A2 provides the corresponding polarization trajectory, which rises early and then declines, offering a complementary view of social stabilization during the transferred run.

The appendix demonstrates that the framework’s *governance layer* transfers together with its control logic. The same communication shield and protocol predicates remain in force in the CybORG loop, so the transfer is not limited to visualization; it is an end-to-end execution of the shielded cyber–social architecture against a different cyber backend. This supports the claim that the framework is modular and extensible.

The transfer results remain limited in scope. The appendix does not establish that V-CHIMERA is optimal for CybORG, nor does it replace the need for a dedicated CybORG benchmark with scenario-specific calibration, more seeds, and comparison against native CybORG baselines. Its narrower contribution is to show that the same coupling abstractions, controller interface, and runtime communication constraints can be preserved when the cyber-defense environment is swapped out.
